# Time-Frequency Representations of Brain Oscillations: Which One Is Better?

**DOI:** 10.3389/fninf.2022.871904

**Published:** 2022-04-14

**Authors:** Harald Bârzan, Ana-Maria Ichim, Vasile Vlad Moca, Raul Cristian Mureşan

**Affiliations:** ^1^Department of Theoretical and Experimental Neuroscience, Transylvanian Institute of Neuroscience, Cluj-Napoca, Romania; ^2^Department of Electronics, Telecommunications and Informational Technologies, Technical University of Cluj-Napoca, Cluj-Napoca, Romania

**Keywords:** neural oscillations, time-frequency representation, machine learning, explainable AI, neurophysiology, electroencephalography

## Abstract

Brain oscillations are thought to subserve important functions by organizing the dynamical landscape of neural circuits. The expression of such oscillations in neural signals is usually evaluated using time-frequency representations (TFR), which resolve oscillatory processes in both time and frequency. While a vast number of methods exist to compute TFRs, there is often no objective criterion to decide which one is better. In feature-rich data, such as that recorded from the brain, sources of noise and unrelated processes abound and contaminate results. The impact of these distractor sources is especially problematic, such that TFRs that are more robust to contaminants are expected to provide more useful representations. In addition, the minutiae of the techniques themselves impart better or worse time and frequency resolutions, which also influence the usefulness of the TFRs. Here, we introduce a methodology to evaluate the “quality” of TFRs of neural signals by quantifying how much information they retain about the experimental condition during visual stimulation and recognition tasks, in mice and humans, respectively. We used machine learning to discriminate between various experimental conditions based on TFRs computed with different methods. We found that various methods provide more or less informative TFRs depending on the characteristics of the data. In general, however, more advanced techniques, such as the superlet transform, seem to provide better results for complex time-frequency landscapes, such as those extracted from electroencephalography signals. Finally, we introduce a method based on feature perturbation that is able to quantify how much time-frequency components contribute to the correct discrimination among experimental conditions. The methodology introduced in the present study may be extended to other analyses of neural data, enabling the discovery of data features that are modulated by the experimental manipulation.

## Introduction

Under normal and pathological conditions, brain circuits engage in rhythmic modulations of activity called neural oscillations (Buzsaki, 2006). Their frequencies span orders of magnitude, from the slow and infra-slow ones, with periods of minutes, tens of minutes, or hours (Aladjalova, 1957; Hughes et al., 2011; Belle and Diekman, 2018; Chrobok et al., 2021), to the medium ones, including theta (4–8 Hz) or alpha (8–12 Hz), the fast ones, like beta (12–30 Hz) or gamma (30–80 Hz) (Mureşan et al., 2008), and, finally, the ultra-fast ones (>100 Hz) (Csicsvari et al., 1999; Hughes, 2008; Gourévitch et al., 2020). These different frequency bands are associated to different brain states. While some specific frequencies also emerge in pathological conditions, like Parkinson’s disease (Foffani et al., 2003), it is an established fact that oscillations are notably expressed in healthy subjects. For example, slower frequencies are associated with sleep (Steriade and Amzica, 1998; Steriade, 2003), while the higher frequencies seem to support cognition and perception (Keil et al., 1999; Klimesch, 1999; Melloni et al., 2007; Başar, 2013; Barraza et al., 2020). In the mid-range, theta oscillations are pervasive in the hippocampus (Wilson and McNaughton, 1994; Skaggs et al., 1996; Buzsáki, 2002; Lega et al., 2012), while alpha rhythms seem to block sensory inputs at cortical level (Traub et al., 2020). To understand their role in normal or abnormal brain function, these ubiquitous rhythmic processes need to be precisely and reliably quantified in brain signals.

One property of neural oscillations renders their estimation in brain signals particularly difficult. Unlike several natural systems, where oscillatory processes may be sustained over longer periods of time (Stuiver and Braziunas, 1989; Huisman and Weissing, 1999; Arnaut and Ibáñez, 2020), brain oscillations are typically transient: They emerge in bursts, or packets (Tal et al., 2020; Moca et al., 2021), which often span only a few cycles in duration. To evaluate their presence in the signal, such packets need to be localized in both time and frequency, simultaneously, and this is very difficult due to the Heisenberg–Gabor uncertainty principle (Gabor, 1946; Heisenberg, 1985).

The time-frequency localization of a signal is most frequently estimated by using time-frequency representations (TFRs) (Boashash, 2015). TFRs are a very common tool used to hunt for oscillation bursts in neural data. Used extensively in the analysis of electroencephalography (EEG) (Başar et al., 2001; Herrmann and Demiralp, 2005) and local-field potential (LFP) (Wilson and McNaughton, 1994; Skaggs et al., 1996; Buzsáki, 2002) data, TFRs are computed using a plethora of spectral techniques, such as short-time Fourier transforms (STFT), Wigner-Ville distributions (WVD), wavelets, and so on. Depending on the particular methodology, different aspects may be emphasized in the signal, e.g., better temporal resolution or better frequency localization (Moca et al., 2021). In addition, some techniques are less robust to noise, which acts as a distractor, hindering the observation of finite oscillation packets in the data (Moca et al., 2021). Other techniques suffer from the smearing of the packet’s representation in time, from its leakage across frequencies, banding, or so-called cross-terms, which are artifactual components introduced by the method itself (Boashash, 2015). Thus, the particular details of the method can vastly impact the properties of the estimated TFR. Furthermore, even for the same method, one may obtain TFRs with different characteristics. Indeed, some methods have one or more parameters (e.g., window size, number of cycles of the mother wavelet, order of the superlet, etc.) and their choice determines what properties of the data are emphasized or suppressed in the representation.

One of the major issues is that it is not always clear what the “correct” (or best suited) TFR of a signal should be. Each estimation technique yields TFRs that may differ in terms of time or frequency resolution (or both), and is sensitive to some properties of the data (Moca et al., 2021). Here we propose one possibility to more objectively assess the quality of a representation: To determine how much useful information the latter extracts from neural signals.

Unfortunately, determining the information content of a TFR is never an easy task, especially when dealing with feature-rich data such as neural time-series data (e.g., EEG or LFP). This is made even harder because the items humans most readily perceive in various signal representations can display features that may not necessarily be systematically related to the experimental condition. Luckily, tools developed in the past decades in Artificial Intelligence (AI), like machine learning (LeCun et al., 2015) may come to the rescue. Such tools can be helpful to judge how much task- or stimulus-related (class-related) information there is in a given labeled dataset by measuring the prediction accuracy (% of correctly classified samples) of trained classification models on test data (Ciuparu et al., 2020). Here, we show that although the “true” time-frequency representation of a signal is not necessarily known, the “quality” of a certain TFR may be judged with the help of machine learning. We use this tool to determine how informative a TFR is about the underlying experimental conditions. We also show that explainable AI techniques (Došilović et al., 2018; Barredo Arrieta et al., 2020; Linardatos et al., 2020) can be used not only to judge the “quality” of a TFR, but also to discover where in the TFR one can find the informative oscillation packets which enable the discrimination among conditions.

## Materials and Methods

### *In vivo* Recordings

Visual stimuli consisted of either moving bars (protocol “RF”; Figure 2A, top) or drifting sinusoidal gratings (0.11 cycles/deg, at a speed of 1.75 cycles/s, protocol “SRCS”; Figure 3A, top) of 4 different orientations and 8 moving directions in steps of 45°, each presented monocularly 10 times on an LCD monitor (Beetronics) with a resolution of 1,440 × 900 pixels and a refresh rate of 60 Hz.

**FIGURE 1:**
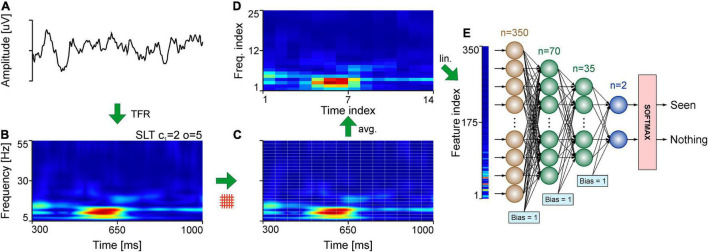
Analysis pipeline for evaluating TFRs. **(A)** Example of single trial EEG signal. **(B)** TFR using the SLT of the EEG signal in panel **(A)**. **(C)** Slicing of the TFR into time-frequency tiles. **(D)** Standardization of the TFR tiles using bicubic interpolation. **(E)** Linearization of the tiled, interpolated TFR in panel **(D)**, fed to the MLP. The example indicates the application of the pipeline for a single trial of the EEG dataset.

**FIGURE 2:**
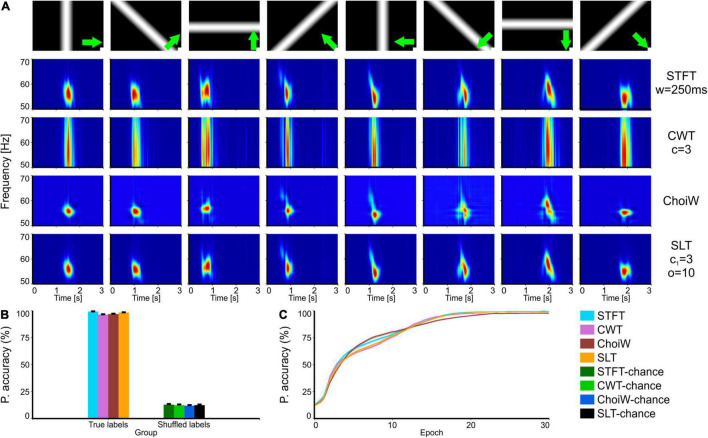
Intracranial electrophysiology with RF visual stimulation protocol. **(A)** Table of TFRs where each column corresponds to a particular grating direction (condition) and each row corresponds to one of the time-frequency analysis methods. **(B)** Average accuracy as measured on the validation set (100 network initializations and dataset splits), with original (true) and shuffled labels (for chance level estimation). **(C)** Learning curves on the validation set for the original labels group (chance level curves were omitted here but reside at ∼12.5%). Error bars are s.e.m.

**FIGURE 3:**
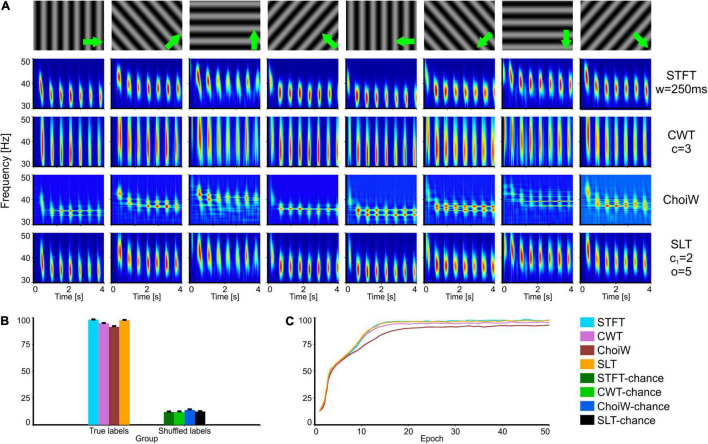
Intracranial electrophysiology with SRCS visual stimulation protocol (SRCS-1). **(A)** Table of TFRs where each column corresponds to a particular grating direction (condition) and each row corresponds to one of the time-frequency analysis methods. **(B)** Average accuracy as measured on the validation set (100 network initializations and dataset splits), with original (true) and shuffled labels (for chance level estimation). **(C)** Learning curves on the validation set for the original labels group (chance level curves were omitted here but reside at ∼12.5%). Error bars are s.e.m.

Adult wild type C57/BL6J mice were anesthetized with isoflurane (5% for induction, 2–2.5% for surgery) and Xylocaine was used as local analgesic. The animals were placed in a stereotaxic frame (Stoelting Co., IL, United States) and the body temperature was maintained at 37°C using a heating pad (Harvard Apparatus). To avoid dehydration during the experiment, saline was infused intraperitoneally (IP). Silicon oil (Sigma-Aldrich) was applied to both eyes to prevent corneal drying and damage. A 2 mm circular craniotomy was performed over the left visual cortex of the animal (2–2.5 mm lateral from midline, 0–0.5 anterior to lambda) to allow insertion of the neural probe. The dura matter was left intact.

Extracellular recordings were acquired using a 32-channel silicon probe (Cambridge NeuroTech) at a sampling frequency of 32 k Samples/s (Multi Channel Systems GmbH) and subsequently band-pass filtered offline (0.1–300 Hz) using a 3rd order Butterworth infinite-impulse response (IIR) filter, applied bidirectionally. All the recordings were downsampled to 1,000 samples/s and a series of notch filters (3rd order Butterworth IIRs, bidirectional) were applied to remove the line noise and its harmonics (50, 100, and 150 Hz). We thus obtained the local-field potentials (LFP) recorded intracortically (Einevoll et al., 2013; Teleńczuk et al., 2017). To minimize animal use, multiple recordings were collected over a period of 6–8 h from each animal.

The data used throughout this study was obtained from three different animals: M048—“SRCS-1,” M60—“SRCS-2,” and M065—“RF.” The channels considered for each analysis were electrodes 19, 11, and 7, respectively.

### Electroencephalography

High density EEG data was digitized at 1,024 samples/second with a 128 electrode Biosemi ActiveTwo machine during a visual recognition task. At the beginning of each trial the subjects were instructed to fixate the center of the screen (baseline) for 1.5–2 s, after which the stimulus was presented on the screen. During the stimulation period subjects were allowed to freely explore the visual scene in order to reach a decision about the identity of the stimulus (object). The stimulation period ended with a key press to signal one of the three possible outcomes: the stimulus was identified (seen), something was visible but the subject was unable to identify it (uncertain), or that no discernable shape was presented on the screen (nothing). A set of 30 shapes comprising familiar objects, fruits, and animals were processed with the “Dots” method (Moca et al., 2011). The trials were organized in 7 blocks with successively increasing visibility to a total of 210 trials per recording session. Visual stimuli (Figure 5A left) spanning 8.7° and 5.6° of visual horizontal and vertical angle, respectively, were presented on a fast response (2 ms) 22-inch LCD monitor (Samsung SyncMaster 226BW). The dots method and the experimental protocols are described in more details elsewhere (Ciuparu and Mureşan, 2016; Bârzan et al., 2021; Moca et al., 2021, 2011). Here, we used data from a single subject, as a testbed to exemplify the TFR evaluation technique.

**FIGURE 4:**
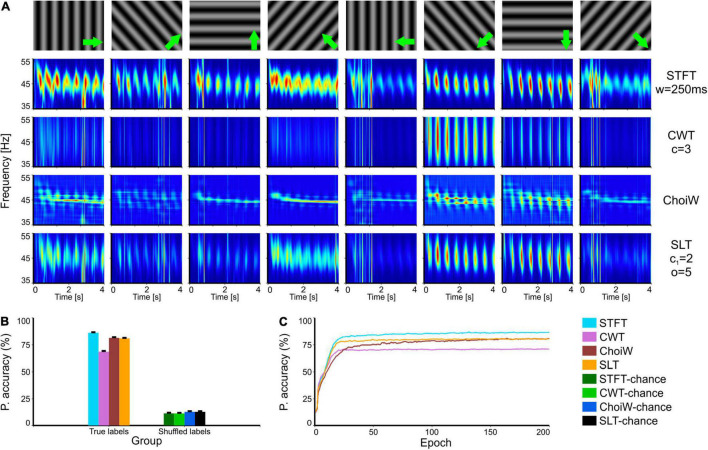
Intracranial electrophysiology with SRCS visual stimulation protocol (SRCS-2). **(A)** Table of TFRs where each column corresponds to a particular grating direction (condition) and each row corresponds to one of the time-frequency analysis methods. **(B)** Average accuracy as measured on the validation set (100 network initializations and dataset splits), with original (true) and shuffled labels (for chance level estimation). **(C)** Learning curves on the validation set for the original labels group (chance level curves were omitted here but reside at ∼12.5%). Error bars are s.e.m.

**FIGURE 5:**
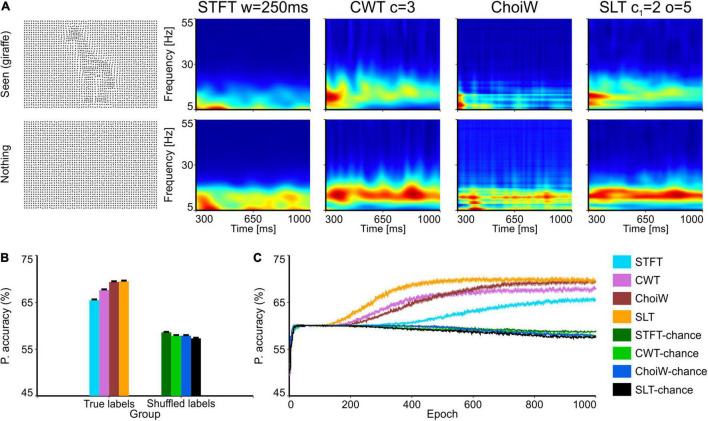
Electroencephalography data with the Dots 30 stimulation protocol. **(A)** Table of TFRs where each row corresponds to the participant’s response (“nothing” or “seen”) and each column corresponds to one of the time-frequency analysis methods. First column on the left shows an example of the visual stimulus for a seen shape (a giraffe, top) and nothing (bottom). **(B)** Average accuracy as measured on the validation set (100 network initializations and dataset splits), with original and shuffled labels for chance level estimation. **(C)** Average learning curves on the validation set. Error bars are s.e.m.

The EEG data, sampled at 1,024 samples/s, was processed offline with Fieldtrip (Oostenveld et al., 2010). First, data was band-pass filtered to 1–150 Hz and two notch filters were also applied to reject the mains noise (50 Hz) and its first harmonic at 100 Hz. Next, channels and trials with abnormally large amplitudes were rejected manually before ICA decomposition. Finally, eye, muscle, and other artifacts were identified and removed from the ICA components and the cleaned EEG was recomposed.

### Short-Time Fourier Transform

The Short-Time Fourier Transform (STFT), also commonly used to compute the *spectrogram*, is one of the most established time-frequency representation methods. The concept behind the STFT is simple and entails computing multiple windowed Fourier transforms and arranging the resulting spectra out in time:


$$S\,T\,F\,T_{x}\,\left(t,f\right)=\int_{-\infty }^{\infty }x\,\left(\tau \right)\,h\,\left(\tau -t\right)\,e^{-j\,2\,\pi \,f\,\tau }\,d\tau$$

where, *x* is the signal of interest and *h* is a time-localizing window function.

Both the time and frequency resolution of the STFT are determined by the length of the analysis window, *h*. A long window favors better concentration in frequency but worse temporal localization, while a short window provides good temporal localization but poor frequency resolution. Therefore, the size of the analysis window is typically set to the time scale of whatever process is being followed in the analysis, but, importantly, this timescale is unique for a given TFR (Moca et al., 2021). Thus, one cannot simultaneously and optimally track multiple timescales using this representation—this is why multiresolution methods are required, such as the continuous wavelet transform or the superlet transform, both described below.

### Continuous Wavelet Transform

The Continuous Wavelet Transform (CWT) is a multiresolution technique that involves convolving the input signal with *wavelets*, which can be thought of as band-pass filter kernels whose scale is adjusted for whatever frequency is being measured. Here, we used the CWT with the complex Morlet wavelets ψ_*f*,*c*_ with a known bandwidth which is defined in terms of the number of cycles *c* of the mother wavelet (Moca et al., 2021):


$$\psi_{f,c}\,\left(t\right)=\frac{1}{B_{c}\,\sqrt{2\,\pi }}\,e^{-\frac{t^{2}}{2\,B_{c}^{2}}}\,e^{j\,2\,\pi \,f\,t}$$

where, *t* is time, *f* is frequency, and *c* is the number of cycles of the mother wavelet, and


$$B_{c}=\frac{c}{5\,f}$$

By scaling the wavelet in time, therefore changing its frequency response, one can measure an arbitrary range of frequencies *via* convolution with the input signal:


$$C\,W\,T_{x}\,\left(t,f,c\right)=2\,{\left|\left(\psi_{f,c}*x\right)\,\left(t\right)\right|}^{2}$$

Because each wavelet scales with the frequency it measures, the time resolution of this method (relative to the timescale of processes evolving on different frequencies) is optimal. The CWT suffers from the opposite drawback, however—its frequency resolution degrades as the measured frequency is increased. To improve it, one must increase the number of cycles *c*, but doing so incurs the cost of worse temporal resolution.

### Superlet Transform

The Superlet Transform (SLT) is a wavelet-based time-frequency estimation technique (Bârzan et al., 2021; Moca et al., 2021) introduced to get around the degrading frequency resolution of the CWT for higher frequencies (Moca et al., 2021). By combining multiple representations in a minimum-mean cross entropic sense (by using the geometric average), the Superlet achieves time-frequency super-resolution. The SLT is defined simply as:


$$S\,L\,T_{x,o,c_{1}}\,\left(t,f\right)={\left[\prod_{i=1}^{o}C\,W\,T\,\left(t,f,c_{i}\right)\right]}^{\frac{1}{o}}$$

where, *x* is the signal, *o* is the order of the superlet, and *c*_1_ is the number of cycles of the base wavelet.

The intuitive explanation of the SLT is that wavelets with small temporal bandwidths have very small (close to zero) responses (coefficients) outside of a process’ time window, while larger wavelets will have very small responses outside of the process’ frequency characteristic, thereby the combination of short and wide wavelets is isolating the process in both time and frequency.

### Wigner-Ville and Reduced Interference Distributions

Another technique widely employed in signal processing is the Wigner-Ville distribution (WVD). A full explanation regarding the WVD is outside the scope of this paper, but can be found in Debnath (2002). In short, it is the Fourier transform, expressed in time-frequency coordinates(*t*,*f*), of the signal’s instantaneous autocorrelation function (IAF), expressed in time-lag coordinates (*t*,τ). For a single-component signal (with a single isolated feature in the time-frequency plane), such as a Gaussian atom (combination of a Gaussian with a complex sine wave), the WVD achieves the best possible time-frequency resolution. For multi-component signals (multiple features in time and/or frequency), however, cross-terms arise between the different components visible as ripples in the time-frequency field. Attempts to eliminate these cross-terms have led to the development of Reduced Interference Distributions (RIDs), which use an extra step to apply a smoothing kernel to the representation (they are called ambiguity kernels as they are applied in the ambiguity domain) (Choi and Williams, 1989; Cordero et al., 2018). Unfortunately, the smoothing kernels degrade the resolution of RIDs when compared to WVD.


$$W\,V\,D_{x}\,\left(t,f\right)=\int x\,\left(t+\frac{\tau }{2}\right)\,x\,\left(t-\frac{\tau }{2}\right)\,e^{-j\,2\,\pi \,f\,\tau }\,d\tau$$

Here, we use the Choi-Williams (ChoiW) distribution (Choi and Williams, 1989), a form of RID, to create time-frequency representations of neural data. It has a single additional parameter, σ, which adjusts the smoothing factor of the ambiguity kernel.

### Feature Vector Extraction

Time-frequency representations were computed on a single channel for each trial (8 conditions with 10 trials each for both SRCS and RF protocols, resulting in 80 representations). The parameters for the techniques were loosely set to obtain good representations for the alpha, beta, and gamma frequency bands: (i) STFTs using 250 ms Blackman windows, zero padded to a Fourier window of 2 s and a time step of 1 ms, (ii) CWTs with 3 cycles for the mother wavelet, (iii) SLTs with either a shorter temporal footprint (*c*_1_ = 2, *o* = 5) or a better frequency concentration (*c*_1_ = 3, *o* = 10), and (iv) ChoiW with σ= 3. The electrophysiological (intracranial and EEG) data was analyzed differently, according to the peculiarities of their respective experiments.

For intracranial recordings, the trial length and frequency of interest regions differ depending on the experimental protocol and the frequency characteristics of the responses. For trials with the SRCS paradigm we extracted TFRs spanning 4 s, while for RF it we considered a temporal window of 3 s, matching the difference in trial size for these different stimulation protocols. Between the three mice that were used in this analysis, each had their stimulus response in another frequency range within the gamma band, which we could normalize to about 20 Hz of bandwidth (RF: 50–70 Hz, SRCS-1: 35–55 Hz, SRCS-2: 30–50 Hz).

For EEG recordings, the trial window was chosen to be a 700 ms window (Figure 1A), occurring 300 ms after stimulus presentation to avoid stimulus onset effects. The frequency range was chosen between 5 and 55 Hz (Figure 1B).

Each analysis technique produces time-frequency representations for the given time segment and frequency range, but each of them have their unique peculiarities in achieving this, especially in the digital domain where representations are discrete. For example, the number of bins per Hz in the SLT and CWT is specified by the user, but the number of bins per second will be equal to the sampling rate of the signal, which differs between the intracranial recordings (1,000 samples/s) and the EEG recordings (1,024 samples/s). STFTs can have varying number of bins for both time and frequency axes (as they can be adjusted using the step size and window length, respectively), and the ChoiW’s binning is determined (fixed) by the sampling rate. Considering the diverse range of time and frequency binning of various methods, a standardization was necessary to compare the representations. Therefore, all TFRs used here were resampled from their native bin sizes (Figure 1C) to a single bin size using bicubic interpolation (Figure 1D), a method widely used in image processing. After this resampling step all TFRs have the same time- and frequency ranges and bin sizes (1 ms and 0.5 Hz per time and frequency bin, respectively, unless otherwise specified). The last step involves compacting the obtained spectrum to a more palatable number of features for classification. This entails establishing numbers of features to obtain per second and per Hz. It essentially means downsampling the time-frequency spectra by splitting in tiles and computing the average of each tile (see Figure 1D). The spectra are then linearized and labeled to prepare them for classification (Figure 1E).

### Information Content Estimation Using Machine Learning

Artificial neural networks (ANNs) were used to quantify the information content of the TFRs. The networks used here were multilayer perceptrons (MLPs) with 2 hidden layers, each layer’s size being 20 and 10% of the size of the input layer, respectively (Figure 1E). This network architecture was determined empirically by evaluating multiple different configurations and selecting the simplest one that had a good performance. Importantly, further optimization may be possible for specific neural datasets, e.g., by increasing the depth of the network or the sizes of the hidden layers. Here we refrained from overoptimizing the networks for the different datasets in order to provide a simple and general proof of concept. Importantly however, we found that, for the data presented here, results were robust across a wide range of architectures.

The networks used a novel activation function called Soft++, discussed in Ciuparu et al. (2020). To train the networks, we used the iRPROP+ (Igel and Hüsken, 2000), an improved version of the resilient backpropagation algorithm (Rprop). Because neuroscience datasets typically yield a large number of features but a small number of samples and, in addition, the neural networks have a large number of parameters, this renders such setups prone to overfitting. To deal with this we introduced a mild dropout strategy (Hinton et al., 2012). The full parameters of the ANNs and of the learning algorithm are given in Table 1.

**TABLE 1:** Architecture of MLP networks, properties of datasets, and parameters of the training algorithm.

	Parameter name/Property	Value
MLP achitecture for LFP data		
	Activation function	Soft++, *c* = 30, *k* = 1 (Ciuparu et al., 2020)
	Input layer size	RF: 600, SRCS-1 and 2: 800
	Number of hidden layers	2
	Hidden layer sizes	20% and 10% of input layer size
	Output layer size	8
	Softmax output layer	Yes
	Weight initialization	LeCun normal (LeCun et al., 2012)
	Initial weight range	[−0.1, +0.1]
MLP architecture for EEG data		
	Activation function	Soft++, *c* = 30, *k* = 1
	Input layer size	350
	Number of hidden layers	2
	Hidden layer sizes	20% and 10% of input layer size
	Output layer size	2
	Softmax output layer	Yes
	Weight initialization	LeCun normal
	Initial weight range	[−0.01, +0.01]
LFP datasets		
	Sample dimensionality (size of feature vectors)	RF: 600 (30 time × 20 freq.), SRCS-1 and 2: 800 (40 time × 20 freq.)
	Number of samples	80
	Number of classes	8 (directions covering 360° in steps of 45°)
	Training set size	48
	Validation/Test set size	32
	Sample (feature vector) normalization	Min-Max range globally scaled to [−3.3823, +1.323] across entire dataset
EEG dataset		
	Sample dimensionality	350 (14 time × 25 freq.)
	Number of samples	150
	Number of classes	2 (“seen” / “nothing”)
	Training set size	90
	Validation/Test set size	60
	Sample normalization	Z-scoring each sample
Training parameters		
	Training algorithm	Batch, IRPROP+ (Igel and Hüsken, 2000)
	Δ_0_	0.001/*Fan-in*
	Δ_*min*_	10^–15^
	Δ_*max*_	0.01 for LFP data, 0.001 for EEG data
	η^–^	0.95
	η^+^	1.05
	Dropout probability	10% (Hinton et al., 2012)

To train and test the networks we used datasets consisting of downsampled and linearized TFRs (feature vectors) computed per experimental trial (see Figure 1). For the EEG dataset, this yielded a total of 150 machine learning samples (feature vectors; 60 “nothing” trials and 90 “seen” trials), while for LFP datasets it provided 80 machine learning samples (10 trials for each direction—see above). For each dataset and each run of training/testing a network, the set of machine learning samples was split randomly into 60% train and 40% validation (test) samples. Training was only performed using the samples in the training set. Furthermore, training was performed for 1,000 epochs and the best network state during the training process was saved. Due to the small number of samples in our datasets, biased sampling during splitting becomes a problem that needs to be mitigated. To average out effects due to the random splitting, as well as due to the random weight initialization of the networks, all dataset splitting, training, and testing procedures were repeated 100 times for each TFR type (4 in total). Finally, to evaluate chance level (especially for unbalanced datasets), we performed the same splitting/training/testing procedure 100 times and for each TFR type while simultaneously randomizing the labels of the datasets for each run.

### Feature Permutation

To evaluate how much a certain feature or set of features contributes to the classification, we developed a methodology based on feature permutation (Fisher et al., 2019). The idea behind this is to perturb each feature at the input of the ANN and to evaluate how much certain performance metrics degrade (e.g., decrease in accuracy or increase in mean-squared error). This is achieved by permuting the values of that feature between different samples such that the values of the feature no longer match the values of the other features nor the classes they belongs to. As a result, the relation between the values of the feature and class labels becomes a random one. If that feature is important for classification, then it is expected that its permutation leads to a degradation of the performance of the classifier. In addition, the importance of the feature can be quantified by measuring the amount of degradation in the performance of the classifier.

One important problem of this strategy is that the perturbation of a feature may not affect the performance of the classifier if other features are strongly correlated to that feature. Indeed, a set of strongly correlated features provides redundant information, resilient to the disturbance of some of the features in the set. Thus, the set of correlated features has to be considered as an aggregate and perturbed together.

Considering these important aspects, we devised a feature permutation methodology consisting of the following steps. The classifier is first trained using the training set and its performance on the validation set is evaluated. This is the reference performance on the “intact” feature space. Second, the pairwise correlation (Pearson) (Nikolić et al., 2012) is computed between all the features values on the validation set. Third, features are considered one by one for the purpose of applying the permutation strategy. Before permuting the current feature, we determine the set of its correlated features by applying a threshold. Here we considered that features are part of the same set if their correlation exceeded 0.5. Next, instead of permuting only the current feature, permutation is applied to all features in the set. After the permutation, the performance of the classifier is evaluated on the new (perturbed) validation set and compared to the reference performance on the intact validation set. The permutation for each correlated feature set is performed multiple times (here we used 10 permutations), and the average change in performance is computed. Finally, the next feature is considered, its correlated set is computed, and the procedure is repeated until all features have been visited.

The permutation of a certain feature set should lead to a decrease in performance (decrease in accuracy, increase in error) but in some cases it can also lead to an increase in performance. In such a case, the feature set acts as a “distractor” for the classifier. The classification performance would be higher if the distractor feature set would be discarded. Thus, the methodology described here can be used to identify both features that are important for classification and features that act as distractors.

## Results

We next tested the TFR evaluation methodology on both intracranial LFP data recorded in mice and on EEG data recorded in humans. First, we focused on a receptive-field (RF) mapping protocol, where drifting, oriented bars are used to map out the structure of RFs in primary visual cortex neurons (Figure 2A). In this case, the passing of the bars through the RF of the cluster of neurons located at the position of the recording electrode induces vigorous gamma bursting at ∼55 Hz. Importantly, due to the retinotopic location of the cluster of neurons relative to the drifting bar, the bursting appears at different moments throughout the trial. This temporal separation makes it such that the ANN will “perceive” increased inputs on different subsets of neurons in the input layer—as a result, classifiers learn the relationship between the TFRs and the experimental condition very robustly (Figure 2B; performance close to 100%) and in very few epochs (Figure 2C). While the SLT and STFT seem to have a slight advantage, performance is almost saturated for most TFRs. In other words, all representations contain sufficient information to unambiguously identify the direction of the moving bars. For such simple cases it is difficult to say which representation is more useful from an information content perspective—but other criteria may be applied, like the temporal and frequency localization of the gamma burst.

In a second batch of tests, we analyzed data recorded from mouse primary visual cortex when using drifting grating stimuli instead of bars. These stimuli induce periodic gamma bursting that occurs at different latencies for different conditions, as shown by all TFRs (Figure 3A). For this case, the STFT and SLT provide the most informative representations (Figure 3B) and also enable the fastest learning (Figure 3C), followed by the CWT. Interestingly, the ChoiW representation, which appears to subjectively generate the sharpest frequency-resolved representation, has the lowest information content about stimulus identity. This occurs because cross-terms expressed in between the gamma bursts appear to be detrimental to the representation. Indeed, the temporal location of the bursts is important for identifying the orientation of the drifting gratings and the cross-terms of the ChoiW representation interfere with the temporal localization of the bursts.

In a third set of tests, we evaluated the information content of TFRs on data recorded with the same type of stimuli (gratings) but that exhibits more pronounced noise, evident as broadband transients in the TFRs (Figure 4A). In this case, the STFT provides the most informative TFR, probably because its poorer temporal resolution. Indeed, at the other end the CWT provides the least information about the stimuli because it emphasizes better the short-lived broadband noise transients (Figure 4B). In this case, the SLT and ChoiW representations offer a tradeoff, although learning is faster in the case of the SLT (Figure 4C). Thus, methods which are more sensitive to brief transients, like the CWT, provide representations that are less accurate when broadband noise contaminates the spectrum.

Intracranial electrophysiology data, such as the LFP, exhibits in general cleaner spectra than extracranial data, such as the EEG. The reason is that in extracranial signals sources of noise are more prevalent. In addition, data in Figures 2–4 was recorded in anesthetized animals, where cortical dynamics may also be more predictable, and the experiment better controlled in terms of visual input. To have a harder and more realistic testbed, we used EEG data recorded in adult human volunteers. We evaluated how much information various TFRs contain about the conscious perception of one human subject on images of dots representing objects with various degrees of visibility (Moca et al., 2011). Classifiers were thus trained to predict the response of the subject (object not seen, i.e., “nothing,” or object “seen”) (Moca et al., 2011) by using exclusively the TFRs computed on the EEG signals recorded from the occipital electrode (Oz; Figure 5A).

As expected, the case of the EEG data is much more difficult for classifiers, which attain a top average performance around 70% (Figure 5B; chance level is ∼60%, given the class unbalance—90 trials with “seen” response compared to 60 trials with “nothing” response). In this case, the best performance was attained on the SLT representation, where classifiers also learned the fastest (Figure 5C), closely followed by the ChoiW distribution. The worst performer was the STFT, which provided the lowest accuracy and slowest learning curve. Thus, for complex spectra, the more advanced methods provide representations with a net advantage over the classically used TFRs, such as the STFT and the CWT.

The results presented so far indicate that various TFRs provide an informative spectral landscape that is correlated to various degrees with the experimental condition. However, it is not sufficient to determine that a representation is informative—ideally one needs to also find out which components of that representation are important and to what degree. To this end, we developed a methodology that is able to quantify how much certain time-frequency components contribute to the accuracy of the classifiers (see section “Materials and Methods”). The methodology is based on feature permutation (Fisher et al., 2019), an algorithm belonging to the larger class of explainable AI techniques (Došilović et al., 2018; Barredo Arrieta et al., 2020; Linardatos et al., 2020).

To determine what TFR components enable the differentiation of perceptual conditions in the EEG data from Figure 5 (“seen” vs. “nothing” responses), we first computed the TFRs for all the trials and created a fixed random split into train and validation sets for all types of TFR. This ensured that a source of variability was eliminated and that all models were trained and tested on the exact same sets of experimental data.

Using the fixed training set, we trained multiple classifiers on the TFRs computed with different methods and selected the best networks, reaching the following top accuracy on the validation set: SLT: 80%, ChoiW: 78.3%, CWT: 71.7%, and STFT: 70%. Next, we applied the feature permutation technique on sets of correlated features (see section “Materials and Methods”) and computed the increase in mean-squared error (MSE) and the amount of decrease in prediction accuracy. These two quantities provide overlapping, but not identical information. If the increase in MSE is a more “continuous” measure of feature importance, the decrease in prediction accuracy is more non-linear (for example, the degradation of a feature may increase error but not sufficiently to generate a misclassification).

**FIGURE 6:**
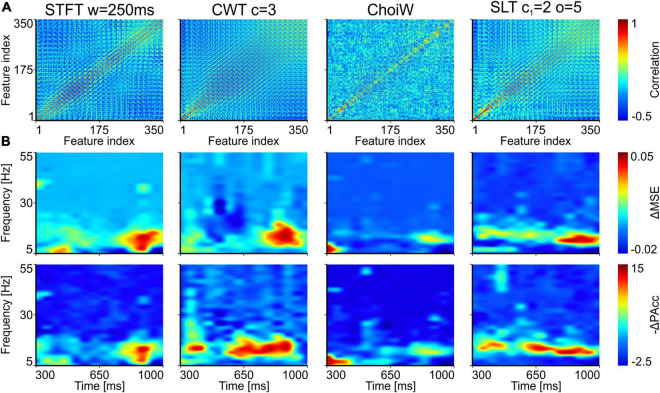
Importance of time-frequency components for distinguishing experimental conditions in the EEG data. **(A)** Pairwise feature correlation for each method. **(B)** Top row: increase in MSE (ΔMSE) when features (time-frequency components) are randomly permuted between trials. Bottom row: magnitude of decrease of prediction accuracy (-ΔPAcc) as a result of feature permutation. Larger positive values represent more important features for correct classification.

Before permuting the features, we computed correlated feature sets that had to be perturbed together, as they provide similar, redundant information (Figure 6A; see section “Materials and Methods”). Interestingly, for the exact same data, different TFRs provided features with different correlation profiles. The most uncorrelated feature space is generated by the ChoiW representation, while the SLT generates a feature space whose correlation profile is in between the STFT and the CWT.

Figure 6B displays the increase in MSE and magnitude of the decrease in prediction accuracy for the different TFRs on the EEG data. In all representations, the time-frequency components that contribute most to distinguishing between “seen” and “nothing” trials are localized around the alpha and low beta frequency bands (8–15 Hz). The clearest picture is provided by the SLT, which also had the highest classification performance on this data. Interestingly, in addition to the alpha band, the prediction accuracy decrease also indicates that a 47 Hz gamma burst, located around ∼400 ms was found useful for classification. For the other representations, the distinguishing TFR components were either less localized in frequency, or scattered more toward the sides of the analyzed time segment. In some cases, the analysis also revealed confounding distractors (negative values in Figure 6B), especially for the CWT and ChoiW. Taken together, these results indicate that the most prominent oscillatory features distinguishing “seen” from “nothing” trials are oscillatory bursts in the alpha/lower-beta bands. This is most clearly evidenced by the SLT representation, which also seems to be the most informative (see Figure 5).

## Discussion

Traditional methods for evaluating the quality of a TFR usually rely on the concept of resolution (Cohen, 1995). In a classical sense, resolution is calculated by estimating the time and frequency uncertainty product of known signals (sine waves, Gaussian atoms). The definition of TFR resolution has been, however, recently challenged (Flandrin et al., 1994; Folland and Sitaram, 1997; Stanković, 2001; Boashash and Sucic, 2003), as this needs to reflect not only the ability to localize an isolated oscillation packet but also the degree to which multiple oscillations can be resolved simultaneously, when they are present in a signal in close temporal and frequency proximity (Moca et al., 2021).

In feature-rich neuroscience data, resolution is not useful for the quantification of the quality of a TFR because the real time-frequency characteristics of the signals cannot be known. The estimations that we use are only reliable insofar as they unravel as many individual components in the signal as possible, while having as little faults (leakage, cross-terms) as possible.

Here, we have shown that, rather than relying on subjective evaluations of how “sharp” a TFR representation is, one can use automatic methods, such as machine learning, which can discover the relation between the features of brain activity, revealed by different representations, and the particular experimental condition. Although time and frequency resolution may be determining factors for the prediction accuracy and learning efficacy, resolution is clearly not the only relevant factor when judging the quality of a TFR. In particular, it is interesting that techniques which are known to provide the best theoretical time-frequency resolution, such as those derived from the WVD, sometimes underperform in terms of information content on neuroscience data. In that sense, machine learning comes to confirm, in an objective manner, previous empirical results (Moca et al., 2021).

Machine learning techniques for the exploration of brain signals have witnessed increased adoption in recent years (Moca et al., 2009; Vu et al., 2018; Glaser et al., 2019; Roy et al., 2019; Li et al., 2020; Lo Giudice et al., 2022). However, it is important to note that such tools have their own pitfalls and may present traps that can mislead even the experimented user. For example, the accuracy reached by a trained neural network model does not depend only on the properties of the data, but also on the particular architecture used, training algorithm, the initial weights, or the random splitting into train and test sets (Ciuparu et al., 2020). Therefore, in general, no conclusion can be drawn on a single trained model but multiple models need to be evaluated. In general, the architecture (often chosen empirically) and training algorithm are fixed for a particular problem, but the impact of the random initial conditions can be averaged out by performing multiple, hundreds or thousands of model instantiations, as we have shown here.

Such multi-model approaches are paramount for datasets encountered in neuroscience, which typically have more features than samples (Ciuparu et al., 2020) and exhibit sample numbers usually in the range of tens to hundreds, at best. Training models on such data is almost always cumbersome. For robust training of machine learning models, especially when using deep learning, hundreds of thousands to millions of samples (Deng et al., 2009) are ideal. In neuroscience, the number of available samples is many orders of magnitude below that. While recording more data is rarely possible, one can use techniques involving multiple random splitting and random network initializations, as well as specialized activation functions that fare well under such difficult conditions (Ciuparu et al., 2020).

Here we have used exclusively MLPs for machine learning, but other options may also be explored. Since TFRs are two-dimensional data representations, it may be interesting to study how convolutional neural networks (CNNs) (LeCun et al., 2015) would fare on such data. There are, however, at least two important aspects to consider. First, CNNs typically require very large datasets to converge, such that convolution kernels can represent the meaningful spatial statistical properties of the data (Krizhevsky et al., 2012). For neural data, it is rarely possible to obtain datasets with even thousands of trials, let alone large datasets with hundreds of thousands/millions of trials. Data augmentation techniques, combined with pooling of datasets across large historical databases may represent a solution. Second, natural images have specific statistical properties (Torralba and Oliva, 2003) that enable CNNs to develop convolution kernels which detect oriented contours, blobs, etc. It is unclear is TFRs possess such universal statistical properties—more likely, TFRs of different types of data may display very different statistics, leading to convolution kernels with very different distributions. In addition, the TFR estimation method is also likely to leave its fingerprint on the statistical properties of the TFR, thereby biasing the learning of the CNN. Nevertheless, exploring the use of CNNs for the evaluation of information content in TFRs is an interesting avenue, worth pursuing in future studies.

The fact that machine learning is able to discover information in neural data is not sufficient for most scientific endeavors. It is equally important to discover how such information is reflected in the properties of the neural data, giving insights into mechanistic brain processes that support perception, cognition, or behavior. As we have shown here, explainable AI techniques (Došilović et al., 2018; Barredo Arrieta et al., 2020; Linardatos et al., 2020) are able to provide insights into why a machine learning model can learn and to indicate where and how relevant information is expressed in the neural data. This is valuable because such automatic techniques may reveal phenomena that the experimenter could not imagine *a priori*.

For the case of neural oscillations, investigated here, our results indicate that different TFRs can reveal different relevant aspects of the data. The informative features of the TFRs we have tested here show remarkable similarity but also slight differences. The SLT emerges as one of the most informative representations, with good concentration of power and less sensitivity to noise. On the other hand, WVD-based techniques or the STFT can also provide informative representations but tend to be less appropriate for signals with a more complex spectral landscape, such as the EEG. In all cases, the explainable AI techniques reveal interesting oscillatory components that largely overlap between representations. However, the same tools also indicate that some TFRs can “catch” informative oscillation packets that the others cannot—and this depends not only on the representation but also on the properties of the data.

To conclude, we have shown that machine learning can be a valuable tool for evaluating the quality of TFRs on various types of neural data. In addition, explainable AI techniques can identify what features of the data are relevant and hint at important neural processes that are difficult to discover without automatic tools. The large-scale adoption of such methods is likely to provide a significant boost to the analysis of neural data in the years to come.

## Data Availability Statement

The original contributions presented in the study are included in the article/supplementary material, further inquiries can be directed to the corresponding author/s.

## Ethics Statement

The studies involving human participants were reviewed and approved by the Local Ethics Committee (1/CE/08.01.2018) and all experiments were conducted in accordance with Directive (EU) 2016/680 and Romanian Law 190/2018. The patients/participants provided their written informed consent to participate in this study. The animal study was reviewed and approved by the Local Ethics Committee (3/CE/02.11.2018), the National Sanitary and Veterinary Authority (approval 147/04.12.2018), performed in accordance with Directive 2010/63/EU and Romanian Law 43/2014.

## Author Contributions

HB, VVM, and RCM designed and implemented the methods. HB, A-MI, and RCM recorded the LFP and EEG data. All authors wrote the manuscript.

## Conflict of Interest

The authors declare that the research was conducted in the absence of any commercial or financial relationships that could be construed as a potential conflict of interest.

## Publisher’s Note

All claims expressed in this article are solely those of the authors and do not necessarily represent those of their affiliated organizations, or those of the publisher, the editors and the reviewers. Any product that may be evaluated in this article, or claim that may be made by its manufacturer, is not guaranteed or endorsed by the publisher.
